# Association of hTERT expression, Her2Neu, estrogen receptors, progesterone receptors, with telomere length before and at the end of treatment in breast cancer patients

**DOI:** 10.3389/fmed.2024.1450147

**Published:** 2024-08-12

**Authors:** Blanca Olivia Murillo-Ortiz, Kenia García-Corrales, Sandra Martínez-Garza, Marcos Javier Romero-Vázquez, Eduardo Agustín-Godínez, Andrea Escareño-Gómez, Daniela Guadalupe Silva-Guerrero, Saulo Mendoza-Ramírez, Mario Murguia-Perez

**Affiliations:** ^1^Unidad de Investigación en Epidemiología Clínica, OOAD Guanajuato, Instituto Mexicano del Seguro Social, León, Mexico; ^2^Servicio de Anatomía Patológica, Hospital General de Zona No. 33, Instituto Mexicano del Seguro Social, Bahía de Banderas, Mexico; ^3^Laboratorio de Anatomía Patológica e Inmunohistoquímica Especializada DIME, Hospital Médica Campestre, León, Mexico; ^4^Departamento de Patología Quirúrgica, UMAE Hospital de Especialidades No. 1, Centro Médico Nacional Bajío, Instituto Mexicano del Seguro Social, León, Mexico; ^5^Servicio de Anatomía Patología, Hospital General de México, Mexico City, Mexico

**Keywords:** hTERT, telomere, telomerase, Her2-neu, immunohistochemistry

## Abstract

**Background:**

Breast cancer shows significant clinical, morphologic, and molecular variation. Telomeres are nucleoprotein complexes composed of hexanucleotide repeat DNA sequence, TTAGGG, and numerous telomere-associated proteins. The maintenance of telomere length is carried out by a ribonucleoprotein called telomerase, which consists of two main components: a catalytic subunit called hTERT (human telomerase reverse transcriptase) and an RNA template called hTR (human telomerase RNA). The importance of evaluating hTERT expression lies in its potential therapeutic application, being an attractive target due to its almost non-existent expression in normal somatic cells. It is also expected that the anti-neoplastic effect would appear earlier in neoplastic cells with shorter telomeres. Additionally, a significant relationship has been observed between Her2-Neu overexpression and Her2-Neu positivity, which could suggest new combined therapies.

The aim of this study was to detect the expression of hTERT, estrogen receptor (ER), progesterone receptor (PR), and HER2-Neu in neoplastic breast tissue embedded in paraffin before treatment and to investigate the relationship between them and with baseline and post-treatment telomere length, as well as with various clinicopathological parameters.

**Materials and methods:**

A cross-sectional-correlational, 21 women diagnosed with breast cancer at the Oncology Service of the High Specialty Medical Unit No. 1 of Bajio of the Mexican Institute of Social Security. The study complies with the Helsinki Declaration and was approved by the Institutional Ethical Committee of the Mexican Institute of Social Security (R-2019-1001-127). A peripheral blood sample was obtained before oncological treatment and at the end of oncological treatment for the measurement of telomere length by extracting DNA from leukocytes, was performed by the quantitative polymerase chain reaction (PCR) method described by Cawthon. Tumor samples were collected from each patient at the oncology department for immunohistochemical determination of biomarker expression (ER, PR, Her2/neu) and hTERT.

**Results:**

Of the 21 cases included in the study, the median age was 57.57 years. Eighteen cases were classified as invasive ductal carcinoma NOS (85.71%), 10 were histologic grade 2 (47.61%), 16 cases were hormone receptor positive (76.19%), 7 were Her2Neu positive (33.33%), and only 2 cases were triple negative (9.52%). Positive hTERT expression was detected in 11 cases (52.38%) and was negative in the remaining cases. A significant association was identified between hTERT-positive cases and Her2-Neu positive cases (*p* = 0.04). Baseline and post-treatment telomere lengths showed a significant difference using the non-parametric Wilcoxon t-test (*p* = 0.002). In hTERT-positive cases, there was significant telomere shortening at the end of oncological treatment (6.14 ± 1.54 vs. 4.75 ± 1.96 Kb, *p* = 0.007).

**Conclusion:**

Positive hTERT immunostaining cases were associated with poor prognostic factors, such as Her2-Neu overexpression and post-treatment telomere shortening. In the future, hTERT immunostaining could be used to select patients for therapies with antagonistic effects on hTERT, as well as in the selection of more appropriate chemotherapy regimens for patients who express it.

## Introduction

Breast cancer shows significant clinical variation due to its morphology and molecular factors. Traditionally, these tumors are classified according to the histological types recognized by the World Health Organization (WHO), and a histological grade is assigned according to the modified Nottingham histological grading system. In all cases without exception, breast cancer biomarkers are performed using immunohistochemistry (IHC) technique (1).

More than 70 years ago, Müller and McClintock established that the ends of eukaryotic chromosomes possess a special structure necessary for maintaining chromosome stability, which they termed “telomeres” (2). Telomeres are nucleoprotein complexes consisting of the DNA sequence of the hexanucleotide repeat, TTAGGG, and numerous telomere-associated proteins, including the six-member Shelterin complex. Their primary function is to protect chromosome ends from chromosomal fusion, recombination, and terminal DNA degradation. Telomeres shorten with each cell replication, continuing until the telomere reaches a critical length, causing cell cycle arrest, leading to senescence or apoptotic cell death. Telomerase primarily consist of two components: a catalytic subunit called hTERT (human telomerase reverse transcriptase) and an RNA template called hTR (human telomerase RNA) (3). Telomerase expression can be measured in tissues or other body fluids in a variety of way, either through the telomere repeat amplification protocol (TRAP), detection of hTERT transcript levels by RT-PCR, or using antibodies for hTERT through immunohistochemistry (4).

The hTERT expression is found only in germ line cells, fetal tissues, and stem cells of healthy tissues; it is not expressed in normal somatic cells or in most benign tumors, except in some large fibroadenomas, where it is highly associated with tumor growth (5).

In most human malignant neoplasms, there is a positive regulation of telomerase, aiding in the survival of cancer cells, leading to differences in both telomere length and telomerase activity between malignant and benign tissues (6). This positive regulation is the major pathway by which telomere length is maintained in humans, and although there are alternative pathways exist, they represent less than 15% (7).

During early carcinogenesis, telomerase expression is low but increases in directly proportion to tumor invasion, preventing cancer cells from entering senescence or apoptosis. This activity is detected in 85–90% of human adenocarcinoma samples, including breast cancer (8). High telomerase activity has been shown to be associated with poor prognosis in breast cancer. This activity is also associated with increased drug resistance in breast and colon cancer cell lines (9).

Telomerase inhibitors are a promising type of therapy aimed at reducing tumorigenicity and suppressing breast cancer growth and metastasis (10). While short-term telomere integrity is not affected when telomerase is suppressed, but chromatin configuration is altered at the histone level, affecting DNA repair and increasing cellular sensitivity to radiation (11).

Similarly, other studies have demonstrated the prognostic value of telomere length, identifying lower survival rates in patients with shorter telomeres (12). Several factors contribute to telomere length in addition to telomerase activity, including environmental and cellular factors that affect telomerase activity and the rate of telomere attrition, as well as genetic characteristics, inherited telomeres, and the number of cell divisions (13).

Hines et al. reported that tumors expressing higher levels of hTERT mRNA were more likely to be histopathologically grade 3, with a high proportion of cells in S phase, and were associated with lymphovascular invasion (14). In contrast, others have suggested that hTERT protein expression is independent of lymph node status, tumor size, and grade, in addition a significant relationship between telomerase activity and positive ER and PR status as contributing factors to telomerase expression, while others claim no such relationship (15). Additionally, the absence of ER beta expression has been associated with low telomerase activity (16). It has been proposed that mutations in the Her2/neu oncogene could induce TERT gene expression, and a significant relationship has been observed between high levels of hTERT and high levels of Her2/neu, suggesting potential for new combined therapies (17).

The aim of our study is to evaluate the expression of hTERT, ER, PR, and Her2/neu in neoplastic breast tissue embedded in paraffin prior to treatment, and to investigate their potential relationships with telomere length both before and after treatment, as well as with various clinical-pathological parameters.

## Subjects and methods

We evaluated 21 women diagnosed with breast cancer at the Oncology Service of the High Specialty Medical Unit No. 1 of Bajio of the Mexican Institute of Social Security. The study complies with the Helsinki Declaration and was approved by the Institutional Ethical Committee of the Mexican Institute of Social Security (R-2019-1001-127).

A peripheral blood sample was obtained before oncological treatment and at the end of oncological treatment for the measurement of telomere length by extracting DNA from leukocytes. Tumor samples were collected from each patient at the oncology department for immunohistochemical determination of biomarker expression (ER, PR, Her2/neu) and hTERT.

Tissue samples were fixed in buffered formalin and paraffin embedded. Sections were cut at 5–6 μm thickness and placed on slides previously covered with DL-lysine. One slide was stained with hematoxylin–eosin for analysis and classification by two independent pathologists, using three-tiered nuclear and histological grading in according to the Bloom and Richardson grading system. The other (unstained) sections were deparaffinized and used for immunohistochemistry.

hTERT (Medaysis, clone A6, dilution 1:100) was assessed using automated techniques on the Pathcom Slide Stainer SSI system. The preparations were observed under a Carl Zeiss Axio Imager A2 light microscope. The expression of ER, PR, Her2/neu, and hTERT was evaluated as nuclear positivity; all controls were appropriate.

Immunoreactivity was estimated using the Allred score as previously described. First, a proportion score was assigned representing the estimated proportion of positively-stained tumor cells (0, none).

### Measurement of telomeres

Collection of blood samples and DNA extraction Peripheral blood samples were obtained by venous puncture in BD Vacutainer^®^ tube. Leukocyte DNA was obtained using the standardized salting out technique, and the concentration was evaluated using a Nanodrop 1000 spectrophotometer (Thermo Fisher Scientific, Wilmington, DE). The samples with a purity value of 1.83 were selected to the analysis. The evaluation of telomere length was performed by the quantitative polymerase chain reaction (PCR) method described by Cawthon (18). The telomere of each sample was assessed by T/S ratio with the number of repeated copies of the telomere (T) and the number of copies of a single control gene (S). 36B4u was used as reference control gene, which codes for a ribosomal phosphoprotein, located on chromosome 12. The telomere PCR and the single copy gene 36B4u (S) were performed separately; the number of cycles required for the accumulation of the fluorescent signal was determined in both reactions (Ct). For the telomere PCR, the conditions were: 40 cycles of 94°C for 10 s, 54°C for 30 s; and for 36B4 PCR: 45 cycles of 95°C for 10 s, 59°C for 30 s. All PCRs were performed using the LightCycler® thermocycler (model 1.5) by Roche thermocycler. To evaluate the efficiency of the reaction, we included a standard curve, performed with serial dilutions of reference DNA in each analysis. The sequence of the probe used to amplify the telomere was the following: Forward (5′-CGGTTTGTTTGGGTTTGGGTTTGGGTTT GGGTTTGGGTT-3′), Reverse (5′-GGCTTGCCTTACCCTTACCCT TACCCTTACCCTTACCCT-3′); and the sequence for 36B4u was: Forward (5′-CAGCAAGTGGGAAGGTGTAATCC-3′) and Reverse: (5′- CCCATTCTATCATCAACGGGTACAA-3′). Using the LightCycler^®^ FastStart DNA Master SYBR Green I kit. The T/S ratio was calculated with the following formula: 2Ct ^telomere^/2Ct ^36B4^ – 1 = 2^-ΔCt^. The conversion from T/S ratio to base pairs (bp) was calculated based on comparison of telomeric restriction fragment length form Southern blot analysis T/S ratios using DNA samples from the human diploid fibroblast cell line IMR90 at different population doublings and the formula 3274 + 2413 × T/S. For all procedures, the initial DNA concentration was 35 ng/μL.

## Results

### Clinical and pathologic characteristics of the patients

Twenty-one female patients with a confirmed diagnosis of breast cancer were included. The median age was 57.57 years (range 34 to 79 years). Of the 21 cases analyzed, 18 were classified as IDC-NOS (85.71%), two cases as invasive lobular carcinoma (ILC; 9.52%), and 1 case as neuroendocrine invasive ductal carcinoma (NE-IDC; 4.72%). Among these 21 cases, 10 were histologic grade 2 (47.61%), eight were histologic grade 3 (35.09%), 2 were histologic grade 1 (9.52%), and one case was not evaluable (4.76%; Table 1). The patients started oncological treatment for the first time with chemotherapy based on anthracyclines (Epirubicin), cyclophosphamide, docetaxel and radiotherapy.

**Table 1:** Clinicopathologic characteristics of patients.

	Median (Range)	*n* = 21	(%)
**Age in years**	57.57 (34–79)		
**Histologic type**			
Invasive carcinoma of NOS Type		18	85.71
Invasive lobular carcinoma		2	9.52
Neuroendocrine Ductal carcinoma		1	4.72
**Histologic grade**			
Grade 1		2	9.52
Grade 2		10	47.61
Grade 3		8	35.09
Not valuable		1	4.76

**Figure 1:**
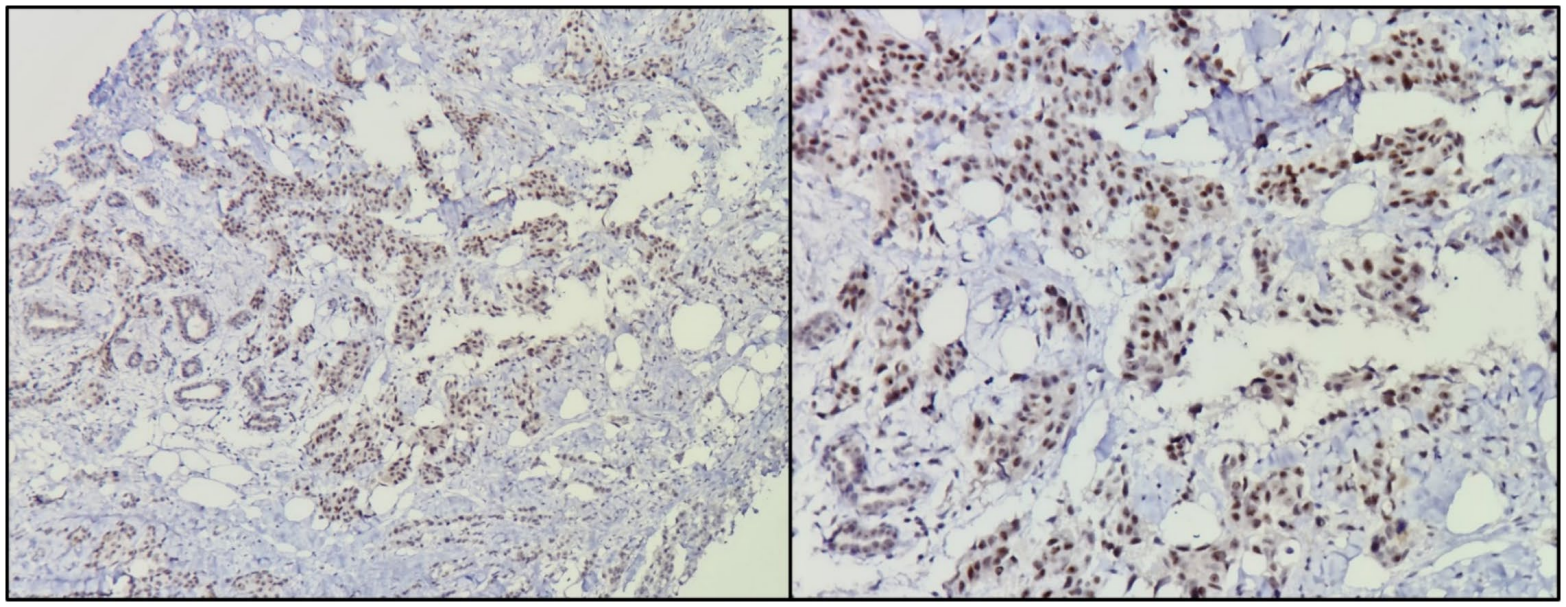
Nuclear expression of hTERT in neoplastic cells. Diaminobenzidine staining in brown. Left, Magnification to 100X. Right, Magnification to 400X.

Immunomarker expression was as follows: 16 cases were positive for hormone receptors (76.19%), seven cases were positive for Her2/neu (33.33%), and only two cases were triple negative (9.52%). Positive expression of hTERT was detected in 11 cases (52.38%; Figure 1), and negative in the remaining 10 cases (47.61%; Table 2.

**Table 2:** Expression of tumor immunomarkers in 21 patients with breast cancer.

	ER		PR		hTERT		Her2/neu	
	n	%	n	%	n	%	n	%
Positive	16	76.19	16	76.19	11	52.38	7	33.33
Negative	5	23.8	5	23.8	10	47.61	14	66.66
**Intensity**								
Mild	2	9.52	3	14.28	0	0	–	–
Moderate	6	28.57	7	33.33	6	28.57	–	–
Intense	8	38.09	5	23.8	5	23.8	–	–
Negative	5	23.8	5	23.8	10	47.61	–	–

ER, Estrogen receptors; PR, progesterone receptors; hTERT, human telomerase reverse transcriptase; Her2/neu, human epidermal growth factor receptor 2.

### hTERT expression and its relationship with immunomarkers: estrogen receptors, progesterone receptors, and Her2Neu

hTERT was found to be present in 52.28% of the patients. No statistically significant relationship was observed between hTERT and histological grade. Using the Mann–Whitney U test to compare the mean age between hTERT-positive and hTERT-negative cases, no statistically significant difference was found (*p* = 0.86; Table 3).

**Table 3:** hTERT expression and its relationship with histological grade.

hTERT expression	Positive (*n* = 11)	Negative (*n* = 10)
**Histologic grade**		
Grade 1 (*n* = 2)	0	2
Grade 2 (*n* = 10)	6	4
Grade 3 (*n* = 8)	5	3
NV (*n* = 1)	0	1

NV, not value.

When analyzing the relationship between hTERT-positive cases and estrogen receptor-positive cases, no statistically significant relationship was found, nor with progesterone (*p* = 0.54). However, a significant association was found between hTERT-positive cases and Her2-Neu positive cases (*p* = 0.04; Table 4).

**Table 4:** hTERT expression and its relationship with immunomarkers: estrogen receptors, progesterone receptors, and Her2/neu expression.

	hTERT Positive (*n* = 11)	hTERT Negative (*n* = 10)	X 2	*p*-value	Fisher exact test
**Estrogen receptors**					
Positive (*n* = 16)	8	8	0.15	0.69	0.54
Negative (*n* = 5)	3	2			
**Progesterone receptors**					
Positive (*n* = 16)	8	8	0.15	0.69	0.54
Negative (*n* = 5)	3	2			
**Her2-Neu expression**					
Positive (*n* = 7)	6	1	4.68	0.03	0.04
Negative (*n* = 14)	5	9			

### Telomere length in relation to hTERT expression

The telomere length in patients at the time of diagnosis was 6.37 ± 1.16 kb with a range of 3.07–8.43, and the telomere length at the end of oncological treatment was 5.33 ± 1.71 kb with a range of 2.16–8.43 kb. A significant shortening was observed, with a statistically significant difference using the non-parametric Wilcoxon t-test (*p* = 0.002).

We analyzed the telomere length in hTERT-positive cases (*n* = 11), which showed significant shortening at the end of oncological treatment (6.14 ± 1.54 vs. 4.75 ± 1.96 kb, *p* = 0.007). hTERT-negative cases (*n* = 10) did not show significant changes in final telomere length (6.62 ± 0.45 vs. 5.96 ± 1.19 kb, *p* = 0.18; Table 5).

**Table 5:** Telomere length in relation to Her2/neu and hTERT expression.

	Telomere length baseline	Telomere length final	*p*-value
**hTERT**			
Positive (*n* = 11)	6.14 ± 1.54	4.75 ± 1.96	0.007
Negative (*n* = 10)	6.62 ± 0.45	5.96 ± 1.19	0.18
**Her2/neu**			
Positive (*n* = 7)	6.04 ± 1.58	4.81 ± 2.16	0.04
Negative (*n* = 14)	6.53 ± 0.91	5.63 ± 1.41	0.04

### Telomere length in relation to Her2-Neu expression

When analyzing the difference between baseline and final telomere length in relation to respect to Her2/neu expression, a statistically significant difference was shown in both cases, both Her2/neu positive (6.04 ± 1.58 vs. 4.80 ± 2.16 kb, *p* = 0.04) and negative (6.53 ± 0.91 vs. 5.63 ± 1.41 kb, *p* = 0.04; Table 5).

## Discussion

hTERT expression has been detected in more than 85% of human malignant neoplasms, with practically nonexistent expression in normal somatic tissues (19). Therefore, the study of telomerase, as well as other components related to telomeres and their function, are attractive targets for the development of new therapies (20). Additionally, it has been observed that TERT’s effects might have functions independent of its role in telomeres and could contribute to the proliferation of cancer cells through other mechanisms (21). We observed positive expression of the hTERT immunomarker in 52.38% of the cases. Kammori M et al. reported positivity in 81% of the cases in their study (22), and like us, did not find a statistically significant relationship between hTERT positivity and patient age, histologic grade, and ER and PR expression, which is consistent with other studies (23, 24). Interestingly, we found a significant association between hTERT expression and Her2/neu overexpression, as observed by Papanikolaou V et al. and Vageli et al. (25, 26).

Other authors, such as Goueli et al., found that the MAP kinase pathway is possibly the mechanism by which the oncogenes Her2/neu, Ras, and Raf induce hTERT transcription, which would be relevant in future therapeutic strategies related not only to telomerase but also to hTERT promoter expression (27).

Thriveni et al. found shorter telomeres in early-stage cancer cases and elongated telomeres in advanced diseases prior to treatment, contrasting with findings from other studies (28). Kammori et al. found that telomere shortening is associated with cancer progression parameters, including stage III TNM disease, large tumor size, a high number of lymph node metastases, and vascular invasion (29). Ceja et al. reported that telomere shortening is a constant feature in cancer cells with higher invasive potential. They also found that the levels of hTERT protein expressed in breast cancer cell lines are not directly related to the degree of invasiveness, suggesting that this may be due to specific differences in telomerase activity in each cell, as well as the fact that not all hTERT functions are related to telomere elongation (30). In our study, we found a statistically significant difference between baseline telomere length and post-treatment telomere length. However, we did not find a statistically significant relationship between hTERT positivity and telomere length prior to treatment. Nevertheless, significant telomere shortening was observed at the end of oncological treatment in hTERT-positive cases compared to hTERT-negative cases. This could be related to poor prognostic factors and not necessarily to the mechanism of telomere shortening.

In the study conducted by Helal et al., a statistically significant association was found between Her2/neu overexpression and short telomere length prior to treatment (31). In our study, when analyzing the cases with Her2/neu overexpression and telomere length both at baseline and after treatment, we did not find a statistically significant relationship. This contrasts with the behavior observed in hTERT-positive cases, where post-treatment telomere shortening was statistically significant, possibly reflecting an increased cancer proliferation rate.

## Conclusion

Positive hTERT immunostaining cases were associated with poor prognostic factors, such as Her2-Neu overexpression and post-treatment telomere shortening. In the future, hTERT immunostaining could be used to select patients for therapies with antagonistic effects on hTERT, as well as for selecting the most appropriate chemotherapy regimens for patients who express it.

## Data availability statement

The original contributions presented in the study are included in the article/supplementary material, further inquiries can be directed to the corresponding author.

## Ethics statement

The studies involving humans were approved by Comité de Ética en Investigación 10,018. The studies were conducted in accordance with the local legislation and institutional requirements. The participants provided their written informed consent to participate in this study. Written informed consent was obtained from the individual(s) for the publication of any potentially identifiable images or data included in this article.

## Author contributions

BM-O: Formal analysis, Methodology, Supervision, Validation, Visualization, Writing – original draft, Writing – review & editing. KG-C: Conceptualization, Investigation, Methodology, Validation, Writing – review & editing, Writing – original draft. SM-G: Formal analysis, Supervision, Writing – original draft. MR-V: Data curation, Software, Writing – review & editing. EA-G: Data curation, Visualization, Resources, Software, Writing – original draft. AE-G: Data curation, Visualization, Writing – original draft. DS-G: Data curation, Visualization, Writing – original draft. SM-R: Software, Resources, Writing – original draft, Writing – review & editing. MM-P: Conceptualization, Funding acquisition, Investigation, Methodology, Project administration, Resources, Supervision, Validation, Writing – original draft, Writing – review & editing.
